# Oculomotor inhibition markers of working memory load

**DOI:** 10.1038/s41598-024-52518-1

**Published:** 2024-01-22

**Authors:** Oren Kadosh, Kfir Inbal, Hadar Snir, Yoram S. Bonneh

**Affiliations:** 1 School of Optometry and Vision Science, Faculty of Life Sciences, Bar-Ilan University, Ramat Gan, Israel; 2 The Leslie and Susan Gonda Multidisciplinary Brain Research Center, Bar-Ilan University, Ramat Gan, Israel

**Keywords:** Working memory, Short-term memory, Saccades

## Abstract

Involuntary eye movements occur constantly even during fixation and were shown to convey information about cognitive processes. They are inhibited momentarily in response to external stimuli (oculomotor inhibition, OMI), with a time and magnitude that depend on stimulus saliency, attention, and expectations. It was recently shown that the working memory load for numbers modulates the microsaccade rate; however, the generality of the effect and its temporal properties remain unclear. Our goal was to investigate the relationship between OMI and the working memory load for simple colored shapes. Participants (N = 26) maintained their fixation while their eyes were tracked; they viewed briefly flashed colored shapes accompanied by small arrows indicating the shapes to be memorized (1/2/3). After a retention period, a probe shape appeared for matching. The microsaccade rate modulation and temporal properties were analyzed for the memory encoding, maintenance, and retrieval phases. Microsaccade inhibition was stronger when more shapes were memorized, and performance improved when microsaccades were suppressed during maintenance and retrieval. This occurred even though the physical stimuli were identical in number under all conditions. Thus, oculomotor inhibition may play a role in silencing the visual input while processing current stimuli and is generally related to processing time and load.

## Introduction

Eye movements, such as saccades and microsaccades, are known to be affected by transient sensory events. Saccades of all sizes are inhibited in response to abrupt stimulation in a process first termed “saccadic inhibition”^1–4^. More recently, it has been described as a general Oculomotor Inhibition (OMI) effect or “oculomotor freezing”^5^, since it was found to generalize to eye blinks^6,7^, smooth pursuit, catchup saccades during pursuit^8^, and ocular drift (Malevich et al.^9^, Ziv et al.^10^, paper in review). The vast majority of OMI studies are based on microsaccades, which are small fixational saccades with a rate of 2–3 per second^11^. Microsaccades can perform precise exploration^12^ and can even be produced voluntarily^13^, but usually they are involuntary, like spontaneous eye blinks and can occur even when there is nothing to explore, as in many experiments with briefly flashed stimuli (e.g.^14^). In response to transient stimuli, microsaccades are first inhibited, at around 150 ms*,* and their probability decreases, then increases above baseline at around 400 ms after stimulus onset, before returning to baseline^15^. This stereotypical inhibition and rebound pattern of rate modulation is affected by the stimulus properties as well as by attention and expectation^7,16–21^. Perceptual oddballs, auditory or visual, were found to prolong the inhibition^22–25^, whereas increased saliency shortened it, for visual^14^, as well as for auditory stimuli^26^, and also during free viewing when the visual transient is induced by a saccade^14,27,28^.

The OMI is also affected by the higher-level properties of the stimulus. For instance, OMI duration differs in written language (words vs different types of non-words)^29^, and in auditory cognition^24^. The inhibition onset and release are affected by name and face familiarity^30–32^, as well as saccade entrainment to speech segmentation^33^. Taken together, these findings indicate that the OMI phenomenon might play a role in suppressing additional transient input caused by saccades during the processing of the current stimulus. The release of inhibition on a timed basis could serve as a synchronization signal in early visual cortex^34^. This results in a series of self-generated events (microsaccades) that are like “punctuation marks” for cognitive processing. In the current study, we explored this interpretation regarding working memory load.

Working Memory (WM) is a cognitive system that temporarily stores information necessary for complex cognitive processing and manipulation^35^. It was first introduced by Baddeley and Hitch^36^ as a brain system constructed of a central executive and two Short-term memory (STM) slave components: the phonological loop and the visuo-spatial sketch pad. The capacity of working memory has been suggested to be limited to four chunks^37^. To date, only a few studies have examined the relationship between WM capacity and the oculomotor system. WM load has been recently found to reduce the microsaccade rate^38^. Another study reported the suppression of reflexive saccades by WM load during anti-saccade tasks^39^. It has been suggested that eye movements may disrupt spatial working memory^40,41^, since both are linked to spatial attention^17,42, 43^. In contrast, a recent study in monkeys suggested that microsaccades facilitate the maintenance of the visual content of WM^44^. An additional study showed that human participants experienced a reduction in visuo-spatial WM when eye movements were prevented during the encoding and maintenance stages but not in the retrieval stage^45^. The latter evidence suggests that, in addition to precise exploration during the encoding WM stage, microsaccades could enhance spatial and feature-based attention of the memorized objects^17,46^ during the maintenance stage.

Current reports are inconsistent; whereas with a high WM load, the attentional demands may result in an increased effort to maintain fixation and minimize disruptions to the ongoing memory task, microsaccades may play a role in the active maintenance and refreshing of information in WM. We deployed a working memory paradigm in which three simple shapes were presented briefly: one, two, or three of them, indicated by pointing arrows, had to be briefly memorized for delayed recall. In this way, the participants had to perform the sequence of WM encoding, maintenance, and retrieval. Our working hypothesis was that silencing the oculomotor system during these different WM stages is necessary to successfully store the memory. We aimed to test this systematically, including three levels of load and probing of the different stages of the WM process. In addition, we investigated the relationship between the rate of microsaccades during the retrieval period and task performance.

## Methods

### Participants

Overall, 26 participants, ages 20–40, were recruited; all were students from the faculty of life sciences in Bar-Ilan University. All participants had normal or corrected-to-normal vision and were naïve to the purpose of the study, except for the first author. The experiments were approved by the Bar-Ilan University Internal Review Board (IRB) Ethics Committee. All participants gave written informed consent, and all the experiments were conducted according to the IRB guidelines.

### Apparatus

Stimuli were displayed at 0.8 m on a 24*″* Eizo Foris FHD monitor with a 100 Hz refresh rate, using an in-house-developed psychophysical platform (PSY) developed by Y.S. Bonneh. The experiment was conducted in dim light; the screen background was gray with 50 cd/m^2^ luminance. We used a remote eye-tracking device (Eyelink-1000, SR Research), with a sampling rate of 500 Hz, and used a 35 mm lens positioned 0.52 m from the participant’s stabilized head using a chin and forehead rest. All recordings were done binocularly, with analyses done monocularly on data from the left eye, based on our experience with the accuracy of microsaccade detection^6,14, 29^. A standard 9-point calibration was performed before each session.

### Stimuli and procedures

**Figure 1:**
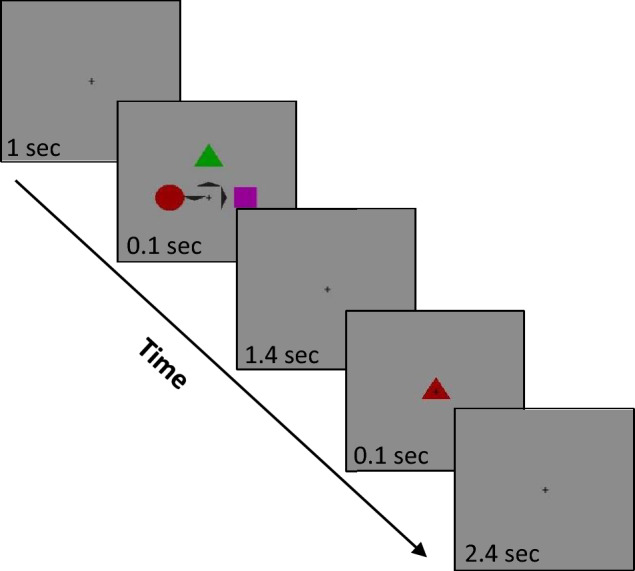
Stimuli and procedure. Participants were instructed to memorize only the items (shape and color) indicated by the dark arrows, and report whether the probed item was identical to one of those items, i.e., the green triangle and the purple square in the example above.

Participants were instructed to maintain fixation and memorize one, two, or three items, shape (three different shapes*,* ~ *1 deg of visual angle, dva*) and color (six different colors), presented around a fixation cross (0.2 dva) for 100 ms*.* The short duration of the stimulus presentation was intended to prevent exploration. The three items were always presented in three fixed locations varying in shape, color, and location, alongside three small dark arrows (0.7 dva) that served as a cue, as illustrated in Fig. 1. The cue could either point to the items that had to be memorized (1,2, or 3) or downwards. After 1.4 s*,* a single colored-shape probe was presented for 100 ms*;* then the participant had 2.4 s to determine and report, using the keyboard, whether the probe was identical to one of the pointed items. Each participant completed three sessions with mixed trials under different conditions, with a total of 48 trials per condition. All shapes and colors were distributed evenly between conditions to prevent a significant luminance difference between conditions. Such a difference could affect the pupil size and might create a bias in microsaccade detection.

### Data analysis

#### Microsaccade and blink detection

For the microsaccade detection, we used the algorithm introduced by Engbert and Kliegl^17^, which is based on eye movement velocity and has been used in our previous studies^14,27–29, 47^. The raw data were first smoothed using the LOWESS method with a window of 15 ms to optimize microsaccade detection, this was found to be useful especially for noisy recordings^17^. A velocity range of 8–150*°*/s and an amplitude range of 0.08–2^o^ were allowed. Eye movements with a duration smaller than 9 ms were also rejected. Eye blinks were detected using an algorithm described in our previous study^6^. We first defined periods with zero pupil size and then extended them by estimating the eyes’ closing and opening time periods, based on the vertical eye movement that typically precedes the blink^29^. Eye tracking epochs were extracted, triggered by the stimulus onset in a range of *− *0.3 to 3 s relative to this trigger with one epoch per experimental trial. Periods of missing data within an epoch, for example, during an eye blink, were discarded from the analysis with an additional margin of 50 ms, without discarding the entire epoch. The rejection rate varied across recordings with a mean of 15% ± 9* SD.*

#### Calculation of the microsaccade rate function

The microsaccade rate modulation function (MS rate) was calculated as described in our previous studies^30,48, 49^, mainly to illustrate the time course of microsaccade occurrences without conducting a statistical analysis. The MS rate was calculated for the raw microsaccade onsets, and it is briefly described here. The rate function was computed for each epoch, by convolving a 500/s rate (the eye-tracker sampling rate) with a Gaussian window with a sigma value of 50 ms at the time of each microsaccade onset (assuming an estimate of one microsaccade per sample duration for a sampling rate of 500 Hz). The rate functions were averaged across the epochs of each participant and condition separately using a 300 ms pre-stimulus baseline correction, and then demeaned by subtracting the participants’ mean. Then, the total average for all conditions and all the participants was added. Finally, the mean and standard error were recalculated across participants.

#### Microsaccade RT calculation

**Figure 2:**
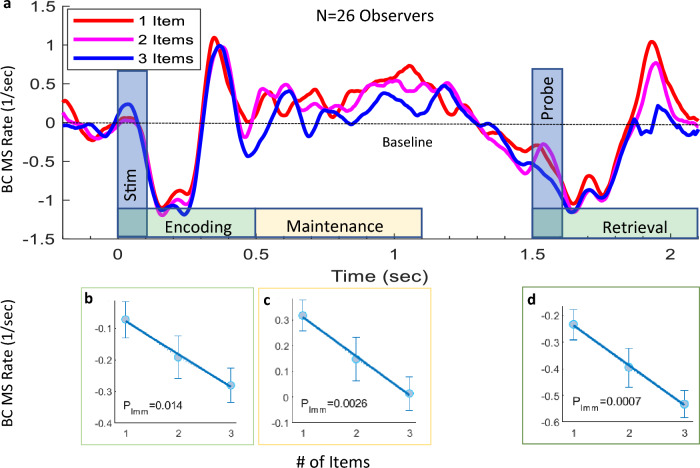
OMI results. (**a**) The event-related microsaccade (MS) rate per second, for illustration purposes, averaged across observers (n = 26) and baseline-corrected (BC) to the pre-stimulus period average, ranging from -300 to 0 *ms*. The stimulus and probe presentation time are denoted by the vertical bars, and the time ranges used for the analyses of the microsaccade (saccades < 2 *dva*) properties are denoted by the horizontal bars. (**b–d**) Baseline corrected MS rate results for each of the different memory stages, all showing a negative relation to the number of memorized items. Significance was assessed using the Linear Mixed Mode (LMM, see the “Methods”) yielding *p* = 0.014 in the Encoding stage (**b**), *p* = 0.0026 in the Maintenance stage (**c**), and *p* = 0.0007 in the Retrieval stage (**d**).

The first microsaccade occurrence relative to stimulus onset, termed the microsaccade reaction time (msRT), was calculated as in our previous studies^32,48, 49^, and determines the end of the inhibition. In addition, to roughly estimate the inhibition onset, we calculated msRT-last, as the last microsaccade before the inhibition onset in a short time window starting at stimulus onset^14^. The msRTs were first averaged for each condition separately within participants and the participants’ mean was subtracted, then averaged across participants. Finally, the total average for all conditions and all the participants was added to restore the ‘real’ values. The error bars were computed across observers on demeaned (within observer) data, with a correction factor (multiplied by √ (n/(n − 1)). This was done to get a better representation of the within-participant effects (Cousineau & Morey’s method^50^; see also^14^). The mean msRT values seemed longer than the MS rate modulation in Fig. 2a illustrates, because we used a long time-range to account for the variance between observers and to obtain a better reliability, since more epochs were included in the calculation.

#### Statistical assessment

**Figure 3:**
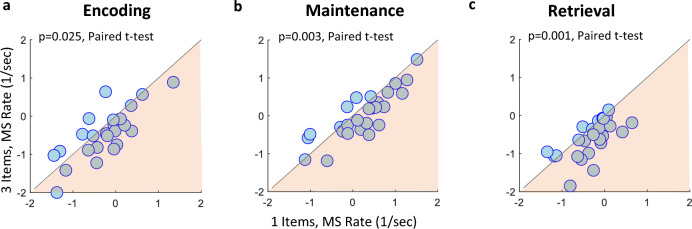
Individual OMI results. Individual differences are depicted through the MS rate (baseline corrected) in a comparison of the two extreme conditions: one-item (X axis) and three-items (Y axis). The results show a higher MS rate for the one-item condition. Significance was assessed using a Paired t-test, yielding *p* = 0.025 in the Encoding stage (**a**), *p* = 0.003 in the Maintenance stage (**b**), and *p* = 0.001 in the Retrieval stage (**c**).

**Figure 4:**
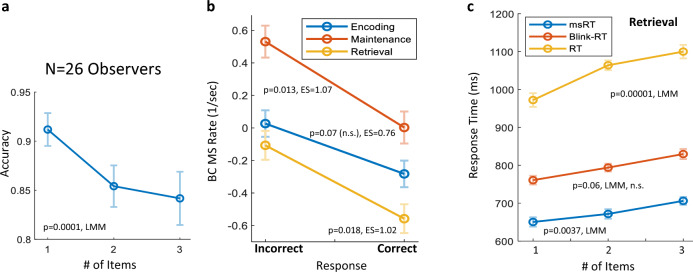
Behavioral and OMI results. (**a**) WM task accuracy results averaged across observers, *p* = 0.0001, Linear Mixed Model (LMM, see the “Methods”). (**b**) BC MS rate comparison, during three time periods, i.e., encoding, maintenance, and retrieval, between correct and incorrect responses, showing that incorrect responses are associated with a higher MS rate, *p* = 0.07*, p* = 0.013* and p* = 0.018, respectively, Paired t-test. The Cohen’s d effect size (ES) was also calculated, yielding *ES* = 0.76*, ES* = 1.07*, and ES* = 1.02, respectively. (**c**) Response times during the retrieval period, measured from the probe onset for the first microsaccade (msRT, see the “Methods”) in blue, the blink RT in orange, and the observer report, and averaged across observers, yielding significance for the msRT and the observer’s RT, *p* = 0.0037* and p* = 0.000001*, LMM*, respectively and a non-significant blink RT.

For the statistical assessment of the linear trends, we used the Linear Mix Model (LMM) analysis^51^. The responses were fitted separately to a simple model of maximum likelihood, with the MS/Blink rate (Fig. 2b–d), Accuracy (Fig. 4a), or Response Time (Fig. 4c) used as the dependent variables, and the number of memorized items set as the predictor variable. For each case, we computed the standard error (SE), the regression coefficient (b), the t-test (t), and the *p*-value of the LMM (p_LMM_) value at a 95% confidence level. In addition, we used paired t-test comparisons of the mean microsaccade rate under different conditions (Figs. 3, 4) and calculated the Cohen’s d effect size (ES).

## Results

In the current study we investigated the relationship between Oculomotor Inhibition and WM load. We collected eye tracking data from 26 participants who performed a working memory task. They had to match a probed item to one to three preceding small colored shaped items, see Fig. 1 for an illustration of the task. We extracted epochs triggered by the stimulus onset in a range of − 0.3 to 3 s relative to this trigger. First, we calculated the microsaccade (MS) rate modulation averaged across participants (*N* = 26), and the baseline corrected to the mean of the 300 ms pre-stimulus period (see the “Methods”).

Figure 2a shows a typical microsaccade inhibition in response to the first stimulus (*time* = 0) during the encoding period; the rate first decreased below baseline (MS inhibition), and then increased above baseline at around 300 ms after the stimulus onset, indicating a release from the inhibition. During the WM maintenance period, the rate stabilized slightly above baseline, showing a profound decrease in spontaneous microsaccade activity in the high WM load condition (blue line). Then, from 1.1 s* onward,* a gradual reduction in the MS rate occurred, reflecting the recruitment of attention in preparing for the probe stimulus^52^. Finally, a second inhibition and release in response to the probe stimulus (*time* = 1.5 s), during the retrieval period, showed a stronger and longer inhibition in the high load condition. The curves exhibited clear periods of MS rate fluctuations, which allowed us to roughly identify time intervals corresponding to the different WM stages. We calculated the MS rate within separate time windows relative to the stimulus onset, 0–0.5, 0.5–1.1, and 1.5–2.1 s, to account for the encoding, maintenance, and retrieval WM stages, respectively. The MS rate results, averaged across observers, show a negative trend, depending on the number of items to memorize, in all three memory stages, reflecting stronger OMI with increasing load. Significance was assessed using the Linear Mixed Model (LMM, see the “Methods”), yielding *p* = 0.014 in the Encoding stage, *p* = 0.0026 in the Maintenance stage, and *p* = 0.0007 in the Retrieval stage, Fig. 2b, c, and d, respectively.

These results were robust and remained significant even when calculated using alternative time ranges. Using longer intervals for the maintenance and retrieval stages yielded *p* = 0.024 with *LMM* in a 0.5–1.5 s period (which lasts until the probe) and *p* = 0.0012 with *LMM* in a 1.5–2.5 s period (which lasts until the average response time), respectively.

Regarding the encoding period, distinction could not have been made between encoding and maintenance related microsaccades during the inhibition-rebound response to the first stimulus and this interval had to be taken as one period. To ensure that the MS rate differences did not stem from eye blinks, we performed a similar analysis, but with the exclusion of trials with blinks in the specific time ranges. The effects remained significant, *p* = 0.034*, p* = 0.0064 and *p* = 0.0027*, LMM* in the encoding, maintenance, and retrieval periods respectively.

In Fig. 3 we plotted Individual data and compared the two extreme conditions of one and three items. The results showed that most observers had a higher MS rate in the one-item condition (red circles), compared with the three-item condition (blue circles) in all three WM stages. *Paired t-test* assessments yielded significance, *p* = 0.025 in the Encoding stage, *p* = 0.003 in the Maintenance stage, and *p* = 0.001 in the Retrieval stage, Fig. 3a, b, and c, respectively.

The task accuracy results, averaged across observers, were above 84% for all the conditions, but were far from a ceiling effect, demonstrating that the task was difficult enough to drive differential microsaccade inhibition. The task accuracy was significantly higher in the one-item condition, *p* = 0.0001, LMM (Fig. 4a). We then looked for a possible relation between microsaccade execution during different memory stages and task accuracy. Figure 4b shows that the MS rate in all three memory stages, i.e., encoding, maintenance and retrieval, was higher for the incorrect responses (14.6 ± 11.1*SD* trials per participant) compared with the correct ones (83.8 ± 22.2*SD*), *p* = 0.07 (*n.s.*)*, p* = 0.013*,* and *p* = 0.018, *Paired t-test*, with a calculated Cohen’s d effect size*, ES* = 0.76*, ES* = 1.07*,* and *ES* = 1.02, respectively, along with one excluded observer who made no mistakes. These results indicate that better WM task performance is associated with a lower MS rate, especially during the maintenance phase.

We plotted the participants’ response times during the retrieval period, measured from the probe onset for microsaccades, blinks, and psychophysics, shown in Fig. 4c. We computed the microsaccade reaction time (msRT, see the “Methods”) as well as the blink RT, as the first microsaccade/blink released from inhibition in a time window ranging from 1.5 to 3 s. The ms/blink RT and the participants’ RT show a positive trend of longer reaction times for more memorized items, *p* = 0.0037*, p* = 0.06 (*n.s.*)*, and p* = 0.00001*,* respectively. The proportion of trials used to calculate the RTs, averaged across all observers and conditions, was as follows: 69% of the trials ± 18 SD for the msRT, 36% of the trials ± 29 SD for the bkRT, and 95% of the trials ± 22 SD for the RT.

**Figure 5:**
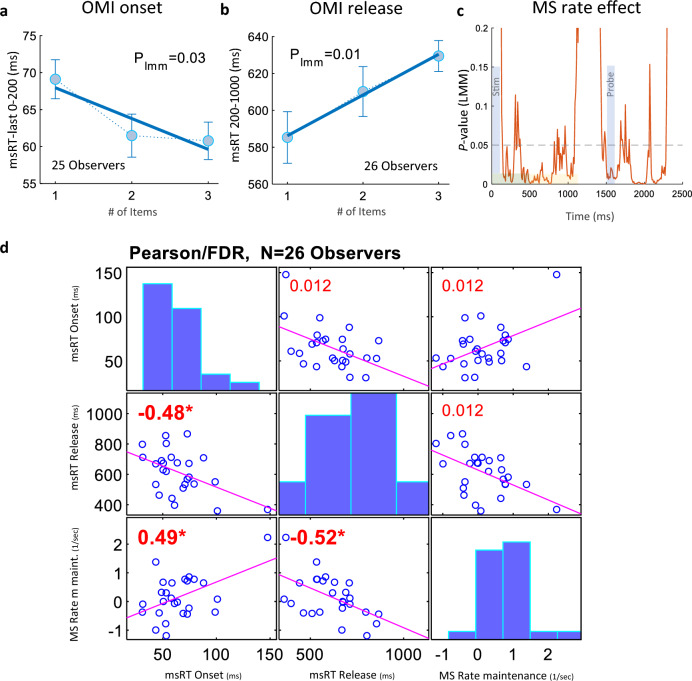
OMI features and MS rate results. (**a**) Inhibition onset time: msRT-last measured here as the last MS latency in the early window of inhibition*, *0–200 ms, for varied WM load (1–3), averaged across observers, *p* = 0.03, Linear Mixed Model (LMM, see the “Methods”). (**b**) Inhibition release time: msRT measured as the first MS latency in the window of release from the inhibition*, *200–1000 ms, for varied WM load, averaged across observers, *p* = 0.01, LMM. (**c**) MS rate effect measured using LMM in a 200 ms sliding window with a 10 ms step size, with the *p-value* denoted in orange, and the light blue bars mark the stimulus onset. The encoding and the maintenance periods are denoted by the horizontal bars. Please note that the x-axis represents the midpoint of the 200 ms time range. (**d**) Pearson’s correlation matrix between the OMI features: onset and release time, and the MS rate measured at a 500–1100 ms time range. The plot shows the *R* (bottom-left) and *p-values* (top-right). All *p-values* were *FDR-corrected*, and all correlations were significant.

We conducted an additional analysis which does not include partitioning to different memory stages. We explored the OMI features in response to the initial stimulus and its relationship with the subsequent MS rate. The OMI onset was calculated as the last occurrence of MS in an early inhibition window of 0–200 ms (see “Methods” for the choice of time window). These results revealed an earlier onset of inhibition for a greater number of items to be memorized, *p* = 0.03*, LMM* (Fig. 5a). The percentage of trials used for this calculation was 13.5% ± 6.8 SD (one observer was excluded for having no MS in this window). This low rate that could be due to temporal anticipation was sufficient to produce a significant memory load effect. The release from MS inhibition was computed as the first occurrence of MS in a time range of 200–1000* ms* and exhibited a positive correlation with the number of items to be memorized, *p* = 0.01*, LMM* (Fig. 5b). Subsequently, for each trial we determined the OMI duration by subtracting the OMI onset msRT from the OMI release msRT.

To assess the robustness of the MS rate results across different time windows, we used a 200 ms sliding window with a 10 ms step size and plotted *p-value* of the Linear Mixed Model statistical test for the effect of the memory load (as in Fig. 2b–d). We observed continuous periods of a significant effect (*p* < 0.05) in 150–290 ms for the initial OMI, then in 400–800 ms presumably in the maintenance period, large periods with *p* < 0.05 during the retrieval, and large segments in the range 0.001–0.01 (Fig. 5c).

Finally, we explored the inter-relations and consistency between the OMI measures of onset and release and the MS rate. The Pearson’s correlation matrix in Fig. 5d demonstrates a moderate correlation between the MS rate and the OMI features: a positive correlation with OMI onset (*R* = 0.49*, p* = 0.012), a negative correlation with OMI release (*R* = *− *0.52*, p* = 0.012), and with a negative correlation between the onset and release (*R* = *− *0.48*, p* = 0.012). The *p*-values were corrected for false discovery rate (FDR)^53^. Given the substantial overlap between the time range selected for the MS rate and the OMI release msRT, some degree of correlation is expected. To address this, we introduced a binary measure termed MS-hit (data not shown), which had a value of one if MS occurred within the MS rate time range and zero for no occurrence; in which case, no clear relationship with the OMI release msRT was expected. Despite this, MS-hit exhibited a negative correlation to the OMI release msRT, even stronger than the MS rate. This may suggest that longer OMI corresponds to a decrease in MS occurrences and thus, lower rate (see also the “Discussion”).

## Discussion

We investigated the effect of the WM load on the oculomotor system, specifically the rate of microsaccades at fixation during the encoding, maintenance, and retrieval memory stages. We expected to find a stronger microsaccade inhibition during the encoding phase and during the memory maintenance phase while making a mental effort to hold the memory of the items and preparing for the probe stimulus. Our results show a reduced microsaccade rate during all memory stages when more items must be memorized (see Figs. 2 and 5c). These results are consistent with a recent study by Dalmaso et al., which found a lower microsaccade rate during the maintenance phase in the high WM load conditions via the retention of more digits or memorizing digits vs. color^38^. However, there are a few differences in the paradigm and analyses. In this recent study, the digits were presented for 1.5 s and the MS rate for the maintenance phase was reported for the inhibition rebound in response to the stimulus disappearance, whereas in the current study the stimulus duration was only 100 ms and the MS rate for the maintenance phase was measured during the period when the stimulus was not present and not in response to the stimulus onset or disappearance.

Moreover, the current results show that correct responses were associated with a lower MS rate in all three memory stages and especially in the maintenance and retrieval phases (see Fig. 4b). Here, with an increasing number of items, we found longer RTs, which typically reflects increased difficulty. This was found for microsaccades as well as blink RTs during the retrieval stage, with a trend similar to the participant’s RT response. In the current study, the cue was presented together with the stimulus for 100 ms. This increased the tasks’ level of difficulty and may have boosted the MS rate effects, specifically during the encoding. Probably with longer presentation times, participants may benefit from making small eye movements towards the items to be memorized. But overall, the task’s difficulty was appropriate for most of the participants, who scored above 80% in all three conditions, as shown by the accuracy results (see Fig. 4a). Previous studies reported a lower MS rate as the task difficulty increased^54^; one study attributed this effect to WM load^55^.

In addition to examining the rate of microsaccades, which can account for the OMI strength, we also measured their timing to account for OMI time-course and duration. The OMI onset after the first stimulus, was influenced by the task; when more items had to be memorized, a faster freezing effect occurred, as evidenced by the last microsaccade occurrence in an early window of inhibition (see Fig. 5a). Furthermore, the first microsaccade occurrence in the late window demonstrated prolonged inhibition as WM load increased (see Fig. 5b). To ensure that the finding of a lower MS rate for increased load was not contingent on our specific choice of time range, we analyzed the significance (*p*-value) of the rate effect using a moving time range, revealing a significant effect of reduced rate for higher load during all memory stages (see Fig. 5c).

Finally, we examined the relation between the transient OMI effects and the later reduction in MS rate. The MS rate during the WM maintenance was correlated positively with the OMI onset and negatively with the release from inhibition (Fig. 5d), i.e., observers with longer OMI duration had lower MS rate relative to baseline during maintenance. We verified that none of the msRT parameters were correlated with the baseline rate to rule out a common source for individual differences (*p* > 0.5). This raises the question whether the sustained reduction of MS rate later than ~ 400 ms post stimulus (Fig. 2a) is part of the transient OMI response, or alternatively a separate byproduct of the WM load in the trial. We suggest that it could be described in analogy to EEG, not as evoked OMI pattern but as an induced inhibition effect, which we interpret as related to the maintenance of the randomly chosen level of WM load in each trial. Taken together, these results indicate that WM load significantly affects both the time-course and strength of the OMI.

It is interesting to compare our results of an earlier onset and delayed release of inhibition under higher WM load (Fig. 5a, b) with previous OMI studies that used different stimuli. Saliency (higher contrast) was found to shorten both the inhibition onset and release (Bonneh et al., 2015, Figs. 2a,b ^14^), while face familiarity was found to shorten the inhibition onset but delay the inhibition release (Rosenzweig and Bonneh, 2019, Fig. 3c,a ^30^), aligning with the present study. We interpret this difference as reflecting the distinction between two different types of processing: (1) low-level effects of stimulus properties as well as early task categorization, which triggers a faster inhibition onset in the case of more items to memorize, and (2) the actual processing of more items that requires longer time, thus prolonging the inhibition.

Although the MS rate was lower than baseline after the first and second stimulus presentations, which typically occurs during abrupt visual stimulation, as well as with auditory stimuli, the MS rate during the maintenance phase, when there was no stimulus, was higher than baseline (see Fig. 2a and c). This may be attributed to spontaneous or ‘‘endogenous” saccades^56^, in contrast to stimulus-dependent or ‘‘exogenous” saccades that occurred during the encoding and retrieval phases in response to stimulus onset. A study that investigated the non-visual gaze patterns, when there was no stimulus, found a reduction in the saccade rate in the WM task, compared with the Long-term memory (LTM) task^57^. It has been proposed that the suppression of microsaccades during target presentation in a visual attention task prevents potential visual disruptions^58^. These reports are consistent with our finding of sparse spontaneous microsaccade occurrences under the high load condition. However, when the stimulus has different spatial locations, the location of the stimulus is also stored^41^ and microsaccades^59^ and saccades^60^ may be directed towards previous spatially attended stimulus locations, suggesting a possible outcome that is opposite to our experiment. In contrast to making saccades in the direction of the stimulus-stored locations, evidence of avoidance or oculomotor inhibition also exists for memorized locations^41^.

There are certain limitations related to the interpretation of our results and the data analyses. In our analyses, we employed time windows that were not defined by clear criteria and sometimes used windows larger than the event region (e.g., inhibition release) depicted in the rate modulation plot. This could potentially lead to overlaps with other windows utilized for different measurements, raising concerns about potential optimization for desired effects. We note that the use of a ‘wide’ window for msRT calculation is intended to adequately sample the distribution (as per our method, epochs without MS within the window are disregarded). However, it’s important to recognize that a larger window may capture a different process. While we have employed this technique in our previous papers with similar window sizes, which increases our confidence, we will now address some critical considerations. (1) The onset of OMI is not expected to occur at time zero due to neural delays, although the MS rate can decrease before the stimulus onset due to anticipation. In our analysis, we estimated the inhibition onset by examining the last microsaccade before the inhibition begins and calculating msRT-last (see the “Methods”) within an early time range of 0–200 ms, as previously done in our studies^14,30^. However, it's important to note that this estimation may be subject to inaccuracies. (2) We selected the 200–1000 ms window for the reasons explained above and to account for the variability in observers' msRTs. Some observers exhibit extended microsaccade latencies, and excluding these msRTs would bias the OMI-release time towards shorter latencies. Nonetheless, the use of a larger time window may introduce potential inaccuracies. (3) The time ranges for the MS rate and OMI release msRT do overlap significantly. Consequently, some degree of artifact correlation can be expected, as longer msRT implies a shorter window for the possibility of additional microsaccades. This assumption is based on the presence of multiple microsaccades within the windows. However, when employing a binary measure (MS-hit, see the Results) unaffected by multiple microsaccades, the correlation persisted, largely mitigating this concern.

To summarize, although spatial covert attention of remembered stimulus locations may drive microsaccades towards the attended locations to enhance memory rehearsal, avoiding making eye movements can reduce memory interference and substitutions potentially increasing memory strength. Our results show earlier and longer OMI and decreased microsaccade rates for higher WM load during all three memory phases, i.e., encoding, maintenance and retrieval, as well as better task performance when microsaccades were suppressed. Taken together, we suggest that maintaining and retrieving more items from working memory is associated with earlier, longer, and stronger oculomotor inhibition. This suggests that event-related OMI is generally related to the associated processing time and load and that OMI may be an indicator of WM load, which is stronger when more items are memorized.

## Data Availability

The experimental datasets generated during the current study will be available from the corresponding author upon reasonable request.
